# Persistence of viral DNA in the epithelial basal layer suggests a model for papillomavirus latency following immune regression

**DOI:** 10.1016/j.virol.2011.03.019

**Published:** 2011-06-05

**Authors:** Gareth Adam Maglennon, Pauline McIntosh, John Doorbar

**Affiliations:** Division of Virology, MRC National Institute for Medical Research, The Ridgeway, Mill Hill, London, NW7 1AA, UK

**Keywords:** ROPV, Papillomavirus, Latency, Laser capture microdissection

## Abstract

Rabbit oral papillomavirus (ROPV) causes benign and spontaneously regressing oral lesions in rabbits, and is a useful model of disease associated with low-risk human papillomavirus types. Here we have adapted the ROPV system to study papillomavirus latency. Following lesion regression, ROPV DNA persists at the majority of regressed sites at levels substantially lower than those found in productive papillomas. Spliced viral transcripts were also detected. ROPV persistence in the absence of disease could be demonstrated for a year following infection and lesion-regression. This was not associated with completion of the virus life-cycle or new virion production, indicating that ROPV persists in a latent state. Using novel laser capture microdissection techniques, we could show that the site of latency is a subset of basal epithelial cells at sites of previous experimental infection. We hypothesize that these cells are epithelial stem cells and that reactivation of latency may be a source of recurrent disease.

## Introduction

Many viruses have a latent stage to their life cycle that is typified by the presence of virus in the absence of clinical signs of disease (Kane and Golovkina, 2010). During latent infection, the production of new virus particles is restricted, and the viral genome is maintained in a silent state with little or no gene expression. Reactivation from latency generally allows the formation of a productive infection, with or without the re-emergence of a visible lesion (Rajcani, 2007). It is considered likely that papillomaviruses form latent infections (Broker et al., 2001; Nicholls et al., 2001; Stubenrauch and Laimins, 1999). The high post-treatment recurrence rates of genital papillomas caused by low-risk HPV types (HPV-6 and HPV-11) suggest that latent infections may occur in genital tissues (Lacey, 2005). HPV-11 and HPV-6 are also responsible for the formation of papillomas in the airways of patients suffering from recurrent respiratory papillomatosis (RRP) (Gissmann et al., 1982; Mounts et al., 1982). RRP is associated with benign papillomas of the larynx, which can recur repeatedly despite surgical intervention. Viral latency has also been proposed as a potential factor responsible for recrudescence, with viral DNA and RNA transcripts often being found in clinically normal tissues of RRP patients (Abramson et al., 2004; Maran et al., 1995; Pignatari et al., 1992; Smith et al., 1993). In addition, there is emerging evidence that HPV may be present in the cervical mucosa in the absence of clinical signs of disease and at low copy numbers, again hinting at the possibility of latent infection (Kalantari et al., 2009).

Studies of animal models of papillomavirus infection have further demonstrated the ability of papillomaviruses to persist in the absence of clinical signs of disease. Infection of the skin of domestic rabbits with cottontail rabbit papillomavirus (CRPV) can induce an asymptomatic infection, characterized by the persistence of viral DNA in the absence of visible signs of disease (Amella et al., 1994; Zhang et al., 1999). Furthermore, asymptomatic infection was shown to be associated with the production of E1 transcripts, which are thought to be required for the stable maintenance of viral genomes in infected epithelial cells. Exposure to exogenous factors such as ultraviolet light could activate the virus leading to the emergence of clinical lesions (Zhang et al., 1999). Most CRPV papillomas do not spontaneously regress, but when complete regression does occur, CRPV DNA can be detected in clinically normal tissues (Selvakumar et al., 1997). Persistence of DNA is at low copy number consistent with only a subset of cells harboring viral genomes. Similarly, it has been suggested that canine oral papillomavirus may form a latent infection with DNA detected following the regression of papillomas in the absence of clinical signs of disease and at low copy number (Stanley et al., 2001).

We chose the infection of domestic rabbits with rabbit oral papillomavirus (ROPV) as a model system to further study papillomavirus latency. ROPV is a naturally occurring infection of domestic rabbits that leads to the formation of benign papillomas in the oral cavity (Parsons and Kidd, 1943; Sundberg et al., 1985). Lesions undergo spontaneous immune-mediated regression and, in contrast to CRPV, have not been reported to undergo neoplastic transformation (Reuter et al., 2001; Syverton, 1952). Therefore ROPV is a more suitable model of low-risk HPV disease than CRPV. Furthermore, unlike COPV and CRPV, ROPV can infect the genital epithelium (Christensen et al., 2000) and has a more similar viral genome and life cycle organization to low-risk HPV types that are thought to have a latent phase such as HPV-11 (Peh et al., 2002). In this study, we have developed the ROPV system to study papillomavirus latency following immune regression. We show that ROPV persists in the absence of clinical and microscopic signs of disease for up to a year following the resolution of disease. The detection of viral DNA and RNA at regressed sites appears to be largely confined to the basal epithelial cells, and is not generally associated with completion of the virus life cycle and the production of new virions. Our results suggest that ROPV genomes are maintained in a latent infection at levels that are compatible with persistence in the basal stem cell. We hypothesize that the virus may be capable of reactivation under certain conditions.

## Results

### Formation of papillomas following experimental infection with ROPV

**Fig. 1:**
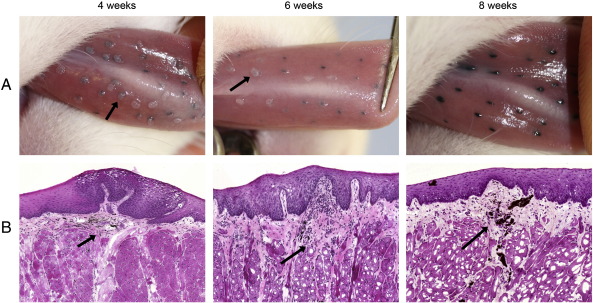
Formation of papillomas following experimental infection. The mucosa of the ventral surface of the tongue was infected using a crude papilloma homogenate mixed with tattoo ink. The gross (row A) and histological (row B) appearance of lesions is shown at 4, 6 and 8 weeks post-infection by photographs taken under general anaesthesia and H&E stained cryosections obtained after culling of animals. The locations of tattoo ink on H&E stained sections are shown by arrows.

Our primary goal was to establish the fate of viral genomes following the immune regression of ROPV papillomas. We therefore sought a means of permanently and accurately identifying areas of the tongue tissue that had previously undergone experimental infection with ROPV, and differentiating them from adjacent regions that had not been infected. A previous study demonstrated that by pin-pricking the undersurface of the tongue with a hypodermic needle and then applying ROPV virions, discrete papillomas measuring 1 mm in size formed at more than 75% of sites (Embers et al., 2002). We modified this technique by pinpricking the tongue with a mixture of ROPV virions and black tattoo ink. Using this modified technique, we also found that visible papillomas formed at 75% of tattoo-marked infected sites but did not appear at uninfected sites. Typically, papillomas were first evident by visual inspection at 2 weeks post-infection. At this time, the tongue was completely healed following the infection procedure and papillomas appeared as small raised and translucent lesions that were associated with no noticeable inflammation. They continued to grow until between 4 and 6 weeks post-infection reaching a maximum size of approximately 2 mm (Fig. 1A). At this time, they had a prominent exophytic appearance with the tattoo mark clearly visible in the underlying tissues. Detrimental effects on general rabbit health, appetite and body condition were not apparent. Around 6 weeks post-infection, immune-mediated regression was apparent, and was associated with a reduction in papilloma size and number, and with complete resolution of papillomas by 8 weeks post-infection. At this time point, the epithelium resumed a normal appearance (other than for the presence of tattoo marks) as determined by gross visual and microscopic examination of tissue sections. We did not observe the formation of visible papillomas at any regions of the lingual mucosa that had not been experimentally infected. Furthermore, papillomas did not form elsewhere within the oral cavity. The location of papillomas was found to correlate very closely with the location of tattoo marks as determined by visual inspection, and tattoo marks were still readily visible as late as a year following experimental infection. Microscopic examination confirmed that tattoo marks were easily visible in the dermis immediately underlying papillomas and that they continued to localize sites of experimental infection beyond the regression of papillomas (Fig. 1B). Addition of tattoo ink did not appear to influence the progression of infection, with identical papillomas forming and regressing concurrently at tattoo-marked and unmarked sites in the same rabbit (data not shown). The tattoo marking technique appears useful therefore, for accurately identifying sites of experimental infection following the regression of papillomas, both by gross inspection and by microscopy.

### Detection of ROPV DNA

**Fig. 2:**
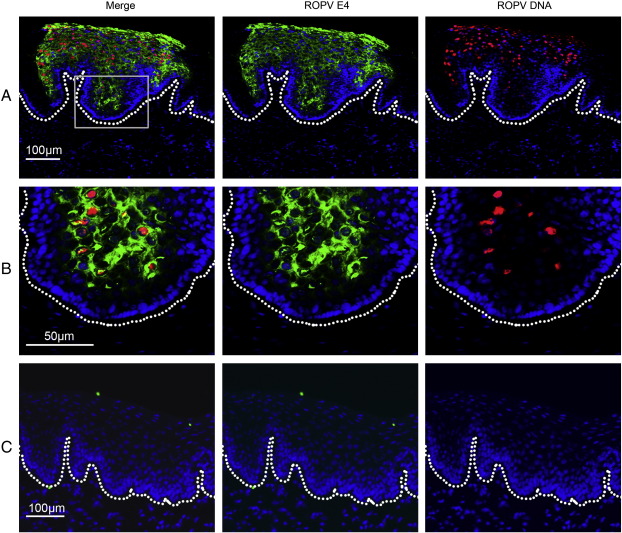
Fluorescent in situ hybridization of ROPV DNA. Presence of ROPV DNA was determined in tissue sections from experimentally infected rabbits using a full-length DIG-labeled probe. Sections were also stained for the presence of ROPV E1^E4 protein. The basal layer of epithelial cells is marked by a dotted line. Fluorescent images are shown for a productive papilloma at 4 weeks post-infection at ×10 (row A) and ×20 (row B). Detection of ROPV DNA generally coincides with expression of E1^E4 protein. ROPV DNA is not visible in the basal layers of the epithelium. At 8 weeks post-infection (row C) following the regression of papillomas, ROPV DNA and E1^E4 protein are no longer visible.

During the productive stages of the ROPV life cycle, ROPV DNA was readily detectable by fluorescent in situ hybridization (FISH). ROPV DNA was first seen in the intermediate to upper layers of the epithelium, and was coincident with the expression of the ROPV E1^E4 protein as determined by indirect immunofluorescence staining (Fig. 2A and B). As found previously, ROPV DNA was not detected in the basal epithelial layers using this approach, (Peh et al., 2002), which most probably reflects the limited sensitivity of the technique. Following papilloma regression, ROPV DNA could not be detected in any of the epithelial layers using in situ hybridization, suggesting that ROPV genome amplification was no longer occurring in the mid to upper epithelial layers, or that it was below the limits of detection using this approach. The failure to detect viral DNA in the basal layers does not rule out its presence at low copy number, but suggested that we would not be able to detect latent ROPV using standard in situ hybridization methods. We decided therefore to use a Laser Capture Microdissection (LCM) approach to determine whether ROPV DNA is indeed present and at what level.

**Fig. 3:**
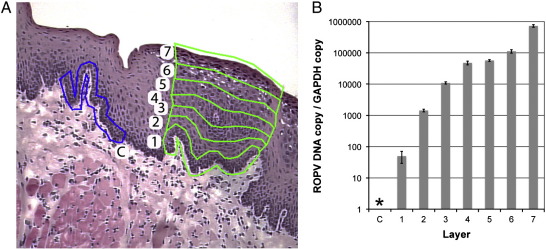
Detection of ROPV in papillomas using LCM. LCM was used to dissect seven (1 to 7) sequential layers of epithelial cells from papillomas harvested at 4 weeks post-infection (A). Uninfected adjacent control samples (layer C) were also obtained. DNA was extracted and real-time PCR performed for quantification of ROPV DNA and GAPDH reference gene. The graph in image B shows a ratio of ROPV copy to GAPDH copy number (with standard error) for each corresponding layer shown in image A. ROPV DNA was not detected in basal layer samples (layer C) taken immediately adjacent to papillomas (*). ROPV DNA was always detected in basal layer samples obtained from papillomas (layer 1). A gradual rise in ROPV DNA copy number was seen between the basal layers and the epithelial surface within papillomas. The degree of amplification was approximately four to five log-fold.

Our LCM technique was optimized on tissue sections taken from rabbits with papillomas, allowing us to quantify DNA copy number during productive infection. Using LCM, papillomas were divided into seven stratified layers from the basal layer to the mucosal surface. Using this approach, ROPV DNA was readily detectable in the basal layers of the epithelium despite the absence of a positive in situ signal (Fig. 3 layer 1). However, we were unable to detect ROPV in the basal layers of the epithelium immediately adjacent to the papilloma (Fig. 3 layer C) consistent with the absence of infection in these tissues. Moving from the basal layers toward the surface of the papilloma, there was a gradual increase in ROPV DNA copy number relative to the glyceraldehyde-3-phosphate dehydrogenase (GAPDH) reference gene, indicative of amplification of viral DNA. The highest levels of ROPV DNA were therefore observed in the uppermost layers of the epithelium and were indicative of a five log-fold amplification of viral DNA compared to the basal-most layers (Fig. 3 layer 7). We consistently detected similar levels of ROPV DNA amplification in the sections obtained from the same papilloma and from other papillomas. Most notably, we failed to detect ROPV DNA in normal epithelium adjacent to papillomas, despite papillomas containing very high levels of viral DNA. This further suggests that following experimental infection, the detection of ROPV genomes was limited only to the infected sites. It appears that the LCM approach is useful in amplifying and detecting ROPV DNA that is below the limits of detection using conventional in situ hybridization methodology.

### Persistence of ROPV DNA following the regression of papillomas

We next sought to determine whether or not ROPV DNA persisted in tongue tissue following the regression of papillomas, possibly as a latent infection. Ten rabbits were infected with ROPV and a further two rabbits served as negative controls. Control rabbits underwent pinpricking and tattooing of the tongue but with the application of phosphate buffered saline in place of ROPV virus suspension. At 4 weeks post-infection, the oral cavities of all animals were thoroughly examined under general anesthesia. The presence of visible papillomas was confirmed in all ten ROPV-infected rabbits, whereas none were visible in the two control animals (Table 1 column 3). Two of the ROPV-infected rabbits (rabbits A and B) were culled at this time point to determine the viral copy number in productive lesions. The remaining rabbits were subsequently examined every 4 weeks to determine whether papillomas were present or absent. Control rabbits did not develop papillomas and were culled at 14 and 24 weeks post-infection. For ROPV-infected animals, all papillomas had regressed by 8 weeks post-infection (as determined by visible inspection) and did not subsequently recur at any of the four-weekly examinations (Table 1 column 4). In this cohort of rabbits, we did not observe spontaneous ROPV reactivation and lesion formation following immune regression.

**Fig. 4:**
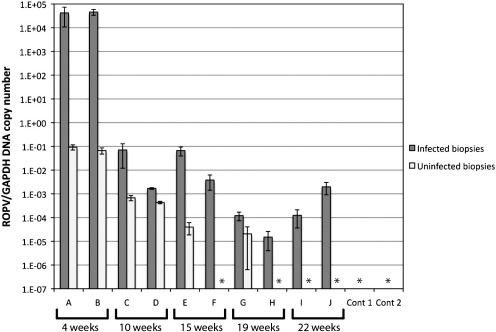
ROPV DNA copy number at infected and uninfected sites. Pairs of rabbits infected with ROPV were culled at successive intervals. ROPV DNA copy number was determined relative to GAPDH at five experimentally infected and five uninfected sites after culling. The average ratio of ROPV DNA to GAPDH reference gene is shown with standard error. Samples marked * indicates that no ROPV DNA was detected in any of the five samples. Two control rabbits underwent scarification but were not infected with ROPV. ROPV DNA was not detected at any of the scarified or non-scarified sites.

Rabbits were culled and tissue samples were dissected from five tattoo-marked ROPV-infected sites, and from five regions of the tongue that had not been experimentally infected to allow quantification of viral DNA by real-time PCR. In tissue biopsies obtained from productive papillomas at 4 weeks post-infection (rabbits A and B), the presence of ROPV DNA was confirmed in all biopsies taken from tattoo-marked ROPV-inoculated sites (Table 1 column 5). ROPV copy number was very high at sites bearing visible papillomas, with 10^4^ to 10^5^ copies of ROPV DNA per copy of GAPDH (Fig. 4). In rabbits with regressed papillomas culled at 10, 15, 19 and 22 weeks post-infection (rabbits C to J) we detected ROPV DNA at the majority (88%) of ROPV-inoculated sites (Table 1 column 5). However, for these rabbits (which no longer bear visible papillomas), ROPV DNA copy numbers were between six and nine log-fold lower at 10^− 5^ to 10^− 2^ copies of ROPV DNA per copy of GAPDH compared to productive infection (Fig. 4). In both rabbits culled at 10 weeks post-infection (rabbits C and D), we could still detect ROPV DNA in tissue samples taken from regions of the tongue that had not undergone experimental infection, although copy numbers were 10 to 100 fold lower than at infected sites (Table 1 column 6 and Fig. 4). It is possible that not all papillomas had undergone complete regression and/or that a low level productive infection may have been present elsewhere in the oral cavity. Beyond 10 weeks post-infection ROPV DNA was only occasionally detected (7% of sites) and copy number was always lower than at equivalent tattoo-marked infected sites from the same rabbit (Fig. 4). From these data, we cannot eliminate the possibility that some of these sites had inadvertently become infected with ROPV either at the time of experimental infection or in the weeks afterward when adjacent papillomas were shedding large amounts of ROPV virions. It is clear however that ROPV can persist at the sites of previous infection long after visible signs of lesion-presence have gone.

### LCM analysis of latently infected tissues

**Fig. 5:**
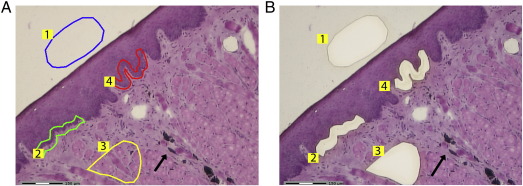
Collection of LCM samples from tissue sections. Sites of experimental infection were identified by means of tattoo marks (arrowheads) on tissue H&E stained tissue sections. The following tissue samples were selected for dissection using LCM (A): blank slide membrane (blue), stromal tissue (yellow), uninfected adjacent basal epithelial cells (green) and basal epithelial cells from immediately above the tattoo mark (red). Tissue samples were collected using LCM into microcentrifuge tubes (B).

Whole tissue analysis allowed us to monitor ROPV persistence following lesion regression. To understand this more thoroughly, we next sought to localize the site of persistence. As tissue biopsies comprise a heterogeneous population of different cell types we focused once more on the laser capture microdissection approach. Between four and eight tattoo-marked tissue sites were identified in biopsy material collected from rabbits culled at 10, 15, 19 and 22 weeks post-infection (rabbits C to J). These were sectioned using a cryostat, and one section from each site was stained with hematoxylin and eosin to establish the integrity of the epithelium. Further sections were subjected to indirect immunofluorescence staining using anti-L1, E1^E4 and MCM7 antibodies. Immunodetection of viral E1^E4 and L1 proteins allowed us to determine whether or not the late stages of the virus life-cycle, and new virion production could be detected post-regression. Detection of the cellular MCM7 protein was used as a surrogate marker for viral E7 protein expression, and was considered to be an indicator of early life cycle events when the protein was present in the suprabasal cell layers. Neither microscopic papillomas nor detectable productive infection was apparent in any of the samples taken from the 10-, 15-, 19- or 22-week rabbits (Table 1 columns 8 & 9). Similarly, there was no evidence of cells in cycle above the basal layers of the epithelium except in the productively infected 4-week rabbits (as shown in Fig. 2). Remaining sections were cut onto PEN membrane-slides, with six adjacent sections placed onto each slide. Basal cells overlying tattoo marks were dissected from all six sections using LCM (Fig. 5) and pooled together for analysis. Thus in total, DNA was extracted from between 1000 and 2000 basal cells. Real-time PCR analysis showed that in a total of 37 tattoo-marked experimentally infected sites taken from seven rabbits, ROPV DNA was present in the basal epithelial cells of 18 (49%) cases (Table 1 column 7). For each of the samples in which ROPV DNA was present in basal cells, three separate control samples were also analyzed (Fig. 5). Pieces of blank PEN membrane adjacent to tongue tissue sections were also dissected. All membrane-control samples were negative for the presence of ROPV DNA (Table 2 column 2), therefore ruling out contamination of slides and reagents with ROPV DNA. Samples of stromal tissue were also dissected from beneath the tattoo-marked sites. The size of the pieces of tissue dissected was always greater in surface area than the size of the corresponding basal cell samples collected. Stromal tissue samples were again always negative for the presence of ROPV DNA (Table 2 column 3), therefore eliminating the presence of contaminating ROPV DNA on the tissue section and ruling out the presence of latent ROPV in the dermal tissues underlying experimentally infected sites. Finally, an equivalent number of basal cells were collected from areas of the same tissue sections that had not been experimentally infected. Of the 18 samples collected, low levels of ROPV DNA were detected in only one (5.6%) of these (Table 2 column 4). ROPV DNA copy number was lower than in the corresponding basal cell sample taken from the experimentally infected site. Given that our control samples eliminated the presence of ROPV DNA contamination on slides and tissue sections, it is possible that a latent infection had been established at this site. A further basal cell sample was obtained from this tissue section and was negative for the presence of ROPV DNA.

ROPV DNA copy numbers in basal cell samples taken from experimentally infected sites were generally very low. The mean and median copy numbers per cell were 81.1 and 6.8 respectively. The high mean value was due to high copy numbers in four samples obtained from rabbits E and H. However, for 83% (15/18) of the samples there was less than one copy of ROPV DNA per cell. This would be consistent with latent genomes being present in only a subset of basal epithelial cells.

Where ROPV DNA was present in basal cells at experimentally infected sites, we also examined the remaining layers of the epithelium using LCM (Fig. 5) to establish whether there was any evidence of genome amplification. For technical reasons (insufficient sample recovery) it was only possible to do this for 13 of the 18 samples (78%). For 12 of these 13 samples (92%) we were unable to detect ROPV DNA in the upper epithelial layers, even though viral genomes were apparent in the basal layer. In these cases, the presence of ROPV in the basal cells did not appear to be associated with the persistence or amplification of DNA in the upper epithelial layers. In one sample however (E-1) copy number per cell was approximately 1000 fold higher in the upper layers compared to the basal cells. Examination of adjacent sections for the presence of L1 and E1^E4 proteins by immunofluorescence and by light microscopy suggested that a productive infection was not present. LCM was also performed on another adjacent section and ROPV DNA was not detected in the upper layers despite being present in basal cells. It may be that amplification of ROPV DNA in sample E-1 was representative of a sporadic productive infection occurring in only a small subset of infected cells.

Occasionally ROPV DNA was detected in the whole tissue biopsies suggesting that a latent infection had formed but ROPV DNA was not detected in basal cell samples analyzed using our LCM approach. It is likely some of these sites did indeed contain viral genomes but below the levels detectable using our methods. Otherwise, our LCM was highly successful in localizing viral genomes to the basal layers of the epithelium at sites of previous experimental infection.

## Long-term persistence of ROPV latency

**Fig. 6:**
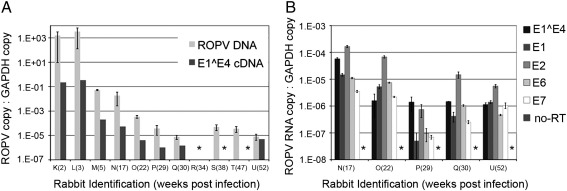
Real-time PCR detection of ROPV DNA and RNA transcripts. ROPV DNA copy number was assessed by real-time PCR relative to GAPDH reference gene in eleven ROPV-infected rabbits. The average ratio of ROPV to GAPDH copy number from five tongue tissue samples is shown with standard error (A). Levels of E1^E4 transcript were also assessed in single tissue samples for each rabbit. Samples marked by * had no ROPV DNA and/or E1^E4 transcripts detected. During latency, E1^E4 spliced transcripts were detected in five rabbits (N, O, P, Q & U). Further RNA analysis was performed on tissues from these rabbits to detect and quantify transcripts originating from the E1, E2, E6 and E7 open reading frames (B). No-RT controls were run for all samples and were always negative (marked *). In addition to detecting spliced E1^E4 ROPV transcripts, RNA sequences originating from the E1, E2, E6 and E7 open reading frames were detected in all of these five rabbits.

The analysis of tissue from this first cohort of rabbits showed that ROPV DNA can persist until at least 22 weeks post-infection, and that viral genomes can be detected 14 weeks after lesion regression. ROPV copy number generally appeared to be higher in rabbits culled at 10 and 15 weeks post-infection as compared to rabbits culled at 19 and 22 weeks post-infection. We therefore sought to determine whether ROPV copy number continued to progressively decline beyond 22 weeks post-infection, and whether latent genomes might eventually disappear altogether. To look at this further, 11 rabbits (rabbits K to U) were infected with ROPV. In addition to our tattoo-marking and infection technique, several tongue sites per rabbit were infected in an identical manner but minus tattoo ink. Three rabbits were culled at 2, 3 and 4 weeks post-infection (rabbits K, L and M respectively) to determine ROPV DNA copy number during productive infection. Subsequently, rabbits were culled at intervals up until 1 year post-infection. Five tattoo-marked experimentally infected sites were harvested from each animal and ROPV DNA copy number determined by real-time PCR (Fig. 6). Again, rabbits were examined at 4 and 8 weeks post-infection to confirm that papillomas had formed and regressed in all animals. Subsequent four-weekly examinations showed that the spontaneous re-emergence of visible papillomas had not occurred. In seven of the eight rabbits culled following papilloma regression, ROPV DNA was detectable at tattoo-marked sites. Importantly, ROPV DNA was still detectable in one rabbit culled at 1 year post-infection (rabbit U). In one rabbit culled at 34 weeks post-infection (rabbit R), ROPV DNA was not detected at any site that had undergone experimental infection with ROPV, despite papillomas being visible at 4 weeks post-infection. It is possible that a latent infection had failed to establish in this rabbit or that latent infection had been effectively cleared by the immune system. Additionally, a tissue sample was taken from the infected but non-tattooed region of the tongue. This showed that where ROPV DNA was detected at tattoo-marked sites, it was also detected at unmarked sites. Therefore presence of tattoo ink did not influence the persistence of viral DNA.

## Detection of ROPV transcription at sites of latent infection

To determine whether or not persistence of ROPV DNA following the regression of papillomas was associated with active transcription of the viral genome, an RT-PCR assay was designed to amplify spliced transcripts spanning the major E1^E4 splice site. The ROPV genome sequence was compared to the HPV 11 genome (Genbank accession numbers AF227240 and NC_001525AF227240NC_001525) and the location of the E1^E4 splice site was predicted. PCR carried out using the E1^E4 primer set on cDNA from productive papillomas (as described in Materials and methods section) identified the splice donor site at nucleotide 1152 and the splice acceptor at nucleotide 3540 within the ROPV genome. The same primers were used for our real-time PCR assay and we confirmed that cDNA but not ROPV genomic DNA was amplified. Using ROPV genomes as template, no PCR product was detected by our real-time PCR assay. Biopsies of experimentally infected tongue tissue were obtained from rabbits in the second time-course experiment (rabbits K to U). As expected, spliced RNA transcripts were detected in abundance during productive infection at weeks two, three and four post-infection (Fig. 6). Following the regression of papillomas, spliced transcripts were still detected in five of the eight rabbits, and most notably were still present at 1 year post-infection in rabbit U. In contrast, no amplification was detected by real-time PCR in uninfected control tissues, “no reverse transcriptase” controls and in no-template controls. Spliced transcripts were not detected in three rabbits culled at 34, 38 and 47 weeks post-infection (rabbits R, S and T respectively). At 34 weeks post-infection (rabbit R), both ROPV DNA and ROPV transcripts were undetectable suggesting that a latent infection was not present. At 38 (rabbit S) and 47 (rabbit T) weeks post-infection, ROPV DNA but not ROPV transcripts were detectable. In these two rabbits, presence of ROPV DNA suggested that a latent infection was present. Failure to detect ROPV RNA may be due to the fact that only a single piece of tissue was available for analysis and a latent infection may not have established at this particular site. When transcripts were detected, their levels showed a broad correlation with ROPV DNA-abundance. For the five post-regression rabbits where spliced ROPV transcripts were apparent (rabbits N, O, P, Q and U), we looked to see whether or not we could also detect RNA sequences encoding the early proteins E1, E2, E6 and E7. Indeed sequences corresponding to E1, E2, E6 and E7 open-reading frames were detectable in all rabbits. Levels of individual transcripts relative to GAPDH varied by less than 100 fold between rabbits (Fig 6B). Similarly, within the same rabbit, levels of different transcripts varied less than 100 fold.

We next sought to demonstrate that the presence of ROPV transcripts were specific to experimentally infected tongue sites and were not detectable at sites that had not undergone scarification. To provide sufficient tissues for this experiment, a further two rabbits were infected with ROPV and culled at 24 weeks post-infection. Five previously infected and uninfected sites were analyzed using our RT-PCR assay for the presence of spliced E1^E4 transcripts for each. Spliced ROPV transcripts were detected at all five experimentally infected sites taken from both rabbits but were not detected in samples taken from uninfected sites. Thus the presence of spliced ROPV transcripts was as expected, specific to tattoo-marked tissues and was not seen in adjacent uninfected tissues.

## Presence of ROPV proteins in latently infected tissues

**Fig. 7:**
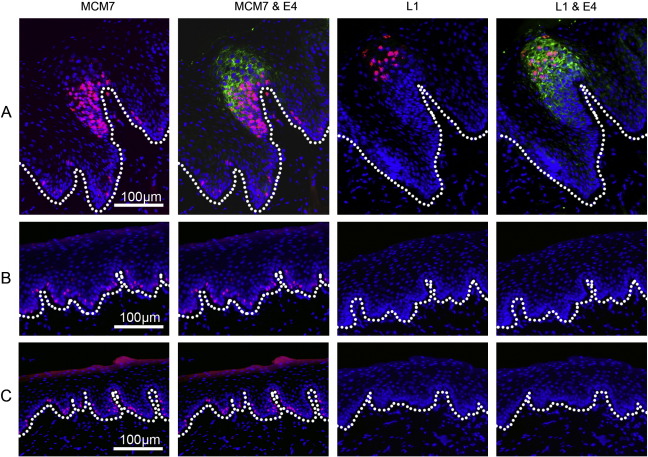
Expression of E1^E4, L1 and MCM7 proteins in ROPV papillomas and latency. Tissue sections were obtained from ROPV papillomas and following regression of lesions during latency. Detection of MCM7, E1^E4 and L1 proteins was performed by immunofluorescence. In ROPV papillomas, expression of MCM7 was present from the basal layer of the epithelium to the mid layers, consistent with expression of E7 protein and maintenance of cells in a proliferative state (panel A). Expression of E1^E4 protein started in the mid layers of the epithelium and overlapped for one or two cell layers with MCM7. Expression was present to the surface of the epithelium and was primarily cytoplasmic in location. Expression of L1 capsid protein was observed in the upper layers of the epithelium consistent with new virion formation and was nuclear in location. Following the regression of papillomas, an extensive analysis of tissue sections failed to demonstrate the presence of E1^E4 and L1 viral proteins (panel B). MCM7 expression was still present in a subpopulation of basal epithelial cells only (panel B) and mirrored that of normal uninfected epithelium (panel C).

Following the regression of papillomas, we did not detect the subsequent re-emergence of visible lesions in any of the rabbits described in this study. Examination of H&E stained tissue sections and sections stained for L1 and E1^E4 from rabbits in the first time course experiment failed to show the presence of microscopic lesions or of a low-level productive ROPV infection (Table 1). However, from our analysis of ROPV DNA and transcripts during latency we cannot discount the possibility that the virus undergoes sporadic activation to form a low-level productive infection. We therefore undertook a more thorough analysis of post-regression tissue sections in order to determine whether or not we could detect ROPV late protein production following papilloma regression by indirect immunofluorescence staining. Four rabbits culled at 17, 22, 29 and 30 weeks post-infection (N, O, P and Q respectively) were selected, from which five tattoo-marked experimentally infected sites were available. Entire tattoo-marked sites were sectioned and half of the sections stained with H&E and the other half analyzed by immunofluorescence using anti-L1 and anti-E1^E4 antibodies. In total, approximately 600 tissue sections were analyzed. Evidence of viral protein expression was not apparent in any of the tissue sections examined, suggesting an absence of microscopic productive lesions (Fig. 7). In comparison, in papillomas harvested at 4 weeks post-infection, there was abundant E1^E4 expression in the mid to upper epithelial layers primarily localized to the cytoplasm of infected cells. In the upper layers of the epithelium toward the epithelial surface, L1 protein was readily detectable. Therefore, in these four latently infected rabbits, the presence of ROPV DNA and transcripts was not associated with a production of new virions as determined by immunofluorescent detection of E1^E4 and L1.

## Discussion

Our previous work has shown that ROPV represents a useful model of low-risk mucosal HPV infection in humans (Peh et al., 2002). Such low-risk HPV types are thought to cause latent infections of the upper respiratory tract in patients suffering from recurrent respiratory papillomatosis (Abramson et al., 2004; Steinberg et al., 1983), and of the genital epithelium in individuals with genital warts (Lacey, 2005; von Krogh et al., 2000). We therefore chose ROPV as a model system to more thoroughly investigate papillomavirus latency. In this study we optimized the ROPV infection procedure to allow the characterization of life cycle events that follow immune regression of papillomas. Our method of tattoo-marking experimentally infected sites has allowed us to accurately identify sites of infection following regression, when clinically detectable disease is no longer present. In all cases, papillomas formed at the site of infection, and always underwent spontaneous regression within an 8-week time frame. Thus ROPV appears to provide a reliable system in which to investigate the phenomenon of papillomavirus latency.

Following ROPV lesion-regression, we did not observe recurrence in any rabbit as determined by visual inspection at regular intervals and thorough examinations of the oral cavity. Thus spontaneous reactivation of ROPV leading to the re-emergence of clinical lesions appeared to be a rare event in these immunocompetent animals. However, in the absence of macroscopic lesions, we could detect ROPV DNA in tissue samples taken from regions of the tongue that had undergone prior experimental infection. Furthermore, we confirmed that the persistence of viral DNA was not associated with microscopic lesions in the epithelium. Similarly, evidence of E1^E4 or L1 expression was not apparent. The continued presence of viral DNA following the regression of papillomas (in the absence of evidence of productive infection) suggested to us that a latent infection had formed. It was also evident that the persistence of viral DNA was not an uncommon event. Evidence of latency was found in the great majority of tissue samples taken from sites that had previously developed papillomas. We failed to detect any residual ROPV DNA at all in only one rabbit. It is possible that in this individual animal, a latent infection did not form at all. Alternatively, a latent infection may have formed but was subsequently eliminated, either by the loss of latently infected cells or by clearance by the host immune system.

Levels of viral DNA detected at latently infected sites were between six and nine log fold lower than at sites bearing visible productive papillomas. The marked differences in viral DNA copy numbers suggest that significant amplification of viral DNA was not occurring as would be expected in a productive ROPV infection. This explains our inability to detect ROPV DNA by in situ hybridization following papilloma regression. Analysis of tissue biopsies also suggested that only a small fraction of the cells contained viral genomes. Because of the heterogeneous nature of the biopsy material however, it was not possible to properly establish copy number in the infected cells. To establish viral genome copy number, our LCM approach was necessary.

Despite confirming the continued presence of ROPV DNA in tissues taken from sites of previous disease, our preliminary analysis did not allow us to establish the particular location of latent infection within the epithelium. To overcome this limitation, we subsequently employed the use of laser capture microdissection. The approach was optimized using tissue sections containing productive papillomas and in these lesions we could readily detect ROPV in the basal layers of the epithelium despite not being able to detect DNA in these cells using fluorescent in situ hybridization. In contrast, we were unable to detect ROPV in the basal epithelial cells of adjacent tissues that had not undergone experimental infection. LCM and real-time PCR provided a means of detecting ROPV DNA in targeted populations of cells and with a sensitivity that we could not achieve using other methods. Using this approach, we were able to detect ROPV DNA in basal cells from around half of the experimentally infected sites at intervals of up to 22 weeks post-infection. Microscopic examination of the tissue sections confirmed that the basal cell ROPV was not associated with either microscopic abnormalities in the epithelium or with the production of detectable levels of late viral proteins.

In the majority of basal cell samples analyzed, there was less than one copy of ROPV per cell, demonstrating that not every cell contained viral genomes. We hypothesize from this that the viral genome may reside as a latent infection in a small subset of basal cells only. It is possible that these “special” cells are epithelial stem cells as suggested previously (Nicholls et al., 2001; Zhang et al., 1999). In contrast, we only once detected a low DNA copy number in a sample taken from an uninfected basal cell sample and not at all in any of the surrounding mesenchymal tissues examined. Interestingly, the presence of viral DNA in the basal layers of the epithelium was typically not associated with viral DNA in remaining upper layers of the epithelium. This suggests that latency can occur without genome amplification in the upper layers of the epithelium, and is supportive of our inability to detect completion of the virus life cycle. In one case however, we did detect a three-log fold amplification in viral DNA upon differentiation; a result which remains to be properly explained. Although this was less than the five-log fold amplification typically seen in productive papillomas, the examination of adjacent tissue sections showed this amplification to be in the absence of microscopic abnormalities and the detection of E1^E4 or L1. Thus, it is possible that occasional and sporadic low level amplification of latent viral DNA may occur in a small number of cells.

We sought to determine whether or not the persistence of ROPV DNA in the absence of clinical lesions was associated with viral transcription. ROPV genome-persistence was associated with production of spliced transcripts spanning the major E1^E4 splice site. Levels of transcripts mirrored the levels of ROPV DNA, and were detectable as late as 1 year post-infection. We detected spliced RNA transcripts in the majority of rabbits tested. Spliced transcripts were specific for experimentally infected sites, indicating that transcripts had not arisen as a result of contamination from elsewhere within the oral cavity. Transcripts were detected at all infected sites examined, suggesting that a low level of transcription was in fact a common occurrence during latency. We also determined the presence of transcripts arising from the E1, E2, E6 and E7 open reading frames. A previous study showed that asymptomatic infection of domestic rabbits with CRPV was associated with production of E1 transcripts and that subsequent activation led to detection of E6 and E7 transcripts (Zhang et al., 1999). Similar findings were made in tissues obtained from patients with HPV-11 associated recurrent respiratory papillomatosis, where in the absence of clinical lesions, E1 and E2 transcripts were detected (Maran et al., 1995). Indeed we also found that both E1 and E2 transcripts were detected in all rabbits examined. However, we also concurrently detected E6 and E7 transcripts in rabbit tissues. These results were not surprising given that E6 and E7 transcripts as well as E1 and E2 transcripts have been shown to be necessary for the stable maintenance of genomes for some papillomavirus types (Oh et al., 2004; Thomas et al., 1999).

Despite detecting low levels of RNA transcripts in latently infected tissues, we did not detect the presence of late viral proteins by immunofluorescence. L1 and E1^E4 proteins were abundantly expressed in the epithelium during productive ROPV infection, but we failed to detect protein in our rigorous analysis of around 600 tissue sections taken from latently infected rabbits. Our findings suggest that ROPV persists in a true latent state without the production of new virions. However, although we did not detect ROPV protein, we cannot completely exclude the occurrence of sporadic instances of productive infection (as suggested by the single finding of high ROPV copy number in the upper layers of one latent infection). It is possible that latent ROPV does occasionally reactivate, but that a low level burst of productive infection ensues that is subsequently suppressed by the immune system. Detection of such bursts would require frequent analysis of tissues from individual rabbits, and possibly also the collection of sequential biopsies. Such analysis was prohibited by the small size of the tongue and the small number of sites that could be infected. Although we did see evidence of apparent ROPV DNA amplification in a tissue sample obtained from one latently-infected rabbit, the analysis of adjacent sections by immunofluorescence failed to demonstrate the presence of L1 or E1^E4 protein.

**Fig. 8:**
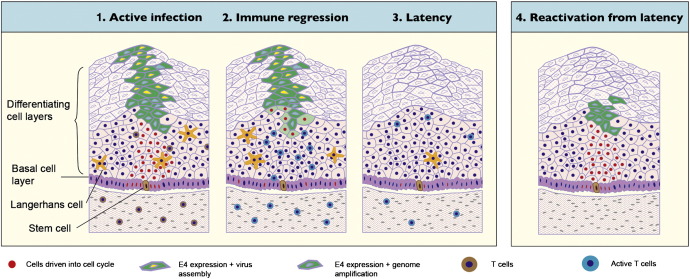
A model of papillomavirus latency following immune regression. An active infection follows entry of papillomavirus into an epithelial stem cell in the basal layer of the epithelium. Cells in the basal layer and above are driven into cell cycle allowing genome amplification and new virion production to occur in the intermediate and upper cell layers. Viral strategies, such as low level protein production in the lower epithelial layers, assist the virus in evading immune detection. Triggering of an effective immune response leads to immune regression, accompanied by infiltration of predominantly T cells. Viral gene expression is shut off and lesion regression occurs. Viral latency may ensue with viral genomes restricted to stem cells in the basal layer of the epithelium. Reactivation of latency is prevented by host immune surveillance. Factors such as immune suppression may allow reactivation to occur. Completion of the virus life cycle may or may not be associated with reappearance of a visible lesion.

In this study, we demonstrate that ROPV can persist at sites of experimental infection following the immune-mediated regression of papillomas. We believe that such persistence is in the form of a latent infection and that subsequent reactivation may be a source of recurrent disease and virus production. It is possible that spontaneous reactivation could occur, although we did not observe this in any of the rabbits in this study. According to our proposed model of papillomavirus latency (Fig. 8), factors external to the cell (e.g. wound healing, hormone levels) may enhance the ability of latent infections to reactivate, and it is likely that the immune system is of central importance to maintaining papillomavirus infection in a quiescent state. Modulation of immunity, for example by induction of immunosuppression, may allow reactivation to occur. We are currently investigating whether or not drug-induced suppression of the adaptive immune system can lead to reactivation of ROPV in experimentally infected rabbits.

## Materials and methods

### Infection of rabbits

All animal experiments were performed in accordance with the relevant legislation in the United Kingdom and with local ethical approval. A crude virus suspension was prepared from ROPV papillomas. Briefly, mature papillomas were harvested from the tongue of a rabbit that had undergone experimental infection with ROPV (a gift of Neil Christensen, Pennsylvania State University, USA) and were homogenized in phosphate buffered saline using an electric rotor-stator homogenizer to form a 10% (w/v) suspension. Virus suspension was mixed 50:50 with black tattoo ink immediately prior to use. Female New Zealand White specified pathogen free rabbits (Harlan Ltd, UK) were anaesthetized using a combination of ketamine hydrochloride (20 mg/kg), buprenorphine hydrochloride (0.05 mg/kg) and medetomidine hydrochloride (0.25 mg/kg) administered by subcutaneous injection. A 23-gauge hypodermic needle was loaded with ROPV by dipping it into the virus suspension. Holding the needle at 90 degrees to the tongue surface, individual punctures were made on the ventral aspect of the tongue. Residual tattoo ink was removed with tissue paper. A further 20 μl of crude virus suspension were added and allowed to “dry in” for several minutes. Anesthesia was partially reversed by administration of atipamezole hydrochloride (1.25 mg/kg) by subcutaneous injection.

### Tissue samples

Animals were culled by intravenous injection of 20% pentobarbitone sodium (200 mg/kg) and the tongue dissected free. Tissues destined for DNA and RNA analysis were dissected using sterile disposable instruments and snap frozen in a mixture of dry ice and isopentane. For tissue sectioning, samples were embedded in OCT compound (CellPath Ltd, UK) prior to snap freezing. DNA was extracted from up to 25 mg tongue tissue using a DNA Micro Kit (Qiagen Ltd, UK) according to the manufacturer's instructions. Total RNA was extracted from up to 100 mg tongue tissue ground to a fine powder under liquid nitrogen using a PureLink RNA Mini Kit (Invitrogen Ltd, UK) according to the manufacturer's instructions. To prepare cDNA from RNA, rigorous DNase treatment of total RNA was first performed twice using Turbo DNA-Free (Ambion Ltd, UK) according to the manufacturer's instructions. Random hexamer primed reverse transcription was then performed using a SuperScript III First Strand Synthesis kit (Invitrogen Ltd, UK) according to the manufacturer's instructions.

### Laser capture microdissection

Tongue tissues were sectioned every 8 μm onto 1 mm PEN membrane slides (Zeiss Ltd, UK) and stained with hematoxylin and eosin. Laser dissection was performed using a PALM MicroBeam system with an ultraviolet laser (Zeiss Ltd, UK). Groups of cells were dissected (typically between 200 and 1000) and captured in 200 μl Adhesive Cap microcentrifuge tubes (Zeiss Ltd, UK). Samples were incubated overnight at 56 °C with 50 μl digestion buffer (10 mM Tris, 1 mM EDTA, 0.1% Tween-20, pH8.0) with 20 μg proteinase K followed by incubation at 95 °C for 10 min. Five microliters of sample were used as template for real-time PCR reactions.

### Detection and quantification of DNA

Real-time PCR was used for the detection and quantification of DNA and cDNA. For the detection of rabbit GAPDH, ROPV genomic DNA and E1, E2, E6 and E7 cDNA, the TaqMan probes and oligonucleotide primers shown in Table 3 were used. Reactions were prepared in a volume of 25 μl containing 1× TaqMan Universal PCR Mastermix (Applied Biosystems Ltd, UK), 40 nM of each primer and 100 nM TaqMan probe. For detection of spliced E1^E4 cDNA, a suitable TaqMan probe could not be designed, therefore SYBR Green chemistry was employed using the primer pair shown in Table 3. Reactions were prepared in a volume of 25 μl containing 1× Absolute SYBR Green QPCR Rox Mix (Applied Biosystems Ltd, UK) with 100 nM each primer. PCR was performed using an ABIprism 7500 system with 15-min denaturation at 95 °C followed by 40 cycles of 95 °C for 15 s and 60 °C for 60 s.

For each real-time PCR assay, a standard curve experiment was performed to allow absolute quantification of DNA. For ROPV genomic DNA and E1, E2, E6 and E7 cDNA, a pUC19 plasmid containing ROPV genome (a kind gift of Neil Christensen, Pennsylvania State University, USA) was used as template. For rabbit GAPDH, a 300 bp fragment of the rabbit GAPDH gene (Genbank accession L23961) was amplified by PCR (forward primer sequence ttcgacaggcagccgcttcttctc, reverse primer sequence ctcggcaccagcatcaccccactt) and cloned into the pDrive vector (Qiagen Ltd, UK) according to the manufacturer's instructions. For the detection of spliced E1^E4 cDNA, a ~ 1200 bp DNA fragment was amplified by PCR from spliced E1^E4 cDNA (forward primer sequence cgccgctgtctttgggggataa, reverse primer sequence aaccggccccgataacgacttgac) and cloned into pDrive vector. Serial dilutions of plasmid DNA were prepared and plots of DNA copy number against cycle threshold value used to determine standard equations. All real-time PCR reactions were run in triplicate along with no-template controls. For determination of cDNA copy number, identical reactions were run minus reverse transcriptase enzyme to control for the amplification of viral DNA and these were always negative. For E1, E2, E6, E7 and ROPV DNA, we determined our assays to be sensitive for the detection of plasmid DNA down to at least eight copies. Our E1^E4 assay was sensitive down to at least 11 copies. Additionally, we demonstrated the sensitivity of our ROPV DNA assay in detecting two copies of ROPV plasmid DNA in rabbit genomic DNA samples extracted from tongue tissue. Efficiency values of real-time PCR amplification were similar for each assay as determined by standard curve analysis. In particular, efficiency values for GAPDH and ROPV DNA were − 3.2 and − 3.27, with a value of − 3.3 indicative of 100% efficiency.

For determination of ROPV DNA in whole tissue biopsies, ROPV DNA copy number was expressed relative to GAPDH copy number. Whole tissue biopsies contained a heterogeneous population of dermal, stromal and epithelial cells many of which are not susceptible to infection with ROPV, therefore a “copy per cell” value would be inaccurate. Similarly, cDNA copy numbers are expressed relative to copy number of GAPDH cDNA. For LCM samples, DNA was extracted from known cell numbers. We determined our real-time PCR assay for GAPDH to amplify 20–30 copies of GAPDH per cell indicating the presence of numerous pseudogenes. Extraction of DNA from samples containing known numbers of peripheral blood leukocytes confirmed that 26 copies of GAPDH were amplified per cell. For LCM samples, a copy number per cell of ROPV DNA was therefore possible.

### Immunofluorescence

Tissues were sectioned every 6 μm on a cryostat and stored at − 80 °C until use. Sections were allowed to warm to room temperature and then fixed in ice-cold methanol (MCM7) or acetone (L1 and E1^E4) for 10 min. Sections were placed in phosphate buffered saline for 5 min and then blocked in 10% fetal calf serum for 20 min. Endogenous peroxidase activity was blocked by incubation of slides in 3% hydrogen peroxide for 6 min. Antibodies were diluted in 3% bovine serum albumin in phosphate buffered saline. Mouse monoclonal anti-L1 (clone K1H8, Dako Ltd, UK) was added at 1:50 and mouse monoclonal anti-MCM7 (Fisher Scientific Ltd, UK) at 1:100 dilution overnight at 4 °C. A biotinylated anti-mouse IgG antibody (Vectorlabs Ltd, UK) was added at 1:100 dilution for 1 h followed by incubation with a streptavidin-biotinylated-HRP complex (Vectastain-ABC, Vectorlabs Ltd, UK) for 30 min according to the manufacturer's instructions. HRP activity was localized and amplified using a Tyramide Signal Amplification Kit (Perkin-Elmer Ltd, UK) according to the manufacturer's instructions and a DAPI nuclear counter-stain applied. For double-staining of E4 protein, a rat polyclonal antibody raised against an ROPV E1^E4 and glutathione-S transferase fusion protein was added at 1:50 dilution for 4 h (Peh et al., 2002). A fluorophore-conjugated anti-rat IgG secondary antibody (Alexa-Fluor 488, Invitrogen Ltd, UK) was added at 1:150 dilution for 60 min.

### In situ hybridization

Tissues sectioned every 6 μm on a cryostat were fixed in 4% paraformaldehyde for one hour at room temperature. Sections were incubated in proteinase K solution (50 μg/ml) for 20 min at 37 °C followed by 3% hydrogen peroxide for 6 min at room temperature. Full length ROPV genomic DNA cloned into a pUC19 vector was labeled with digoxigenin (DIG) using a DIG DNA Labeling Kit (Roche Applied Science Gmbh, Germany) according to the manufacturer's instructions. Labeled probe diluted to 40 pg/μl in hybridization buffer (50% formamide, 5% dextran sulphate, 1× Denhardt's solution, 4× SSC, 200 μg/ml salmon sperm DNA) was added to sections with a cover slip and heated to 95 °C for 5 min followed by cooling on ice for one minute. Hybridization was performed at 42 °C overnight in a humid box. The following day, cover slips were removed and the slides washed for 10 min in formamide wash buffer (50% formamide, 2× SSC, 0.05% Tween-20) followed by 10 min in 2× SSC both at 42 °C. Non-specific binding of antibody was prevented by blocking sections in 10% fetal calf serum for one hour. An anti-DIG HRP-conjugated antibody (Roche Applied Science Gmbh, Germany) was added at 1:400 dilution for one hour. HRP activity was localized and amplified using a Tyramide Signal Amplification Kit (Perkin-Elmer Ltd, UK) according to the manufacturer's instructions and a 4',6-diamidino-2-phenylindole (DAPI) nuclear counter stain added.

## Figures and Tables

**Table 1:** Summary data from infected rabbits.

Rabbit data		Gross examination		Whole tissue biopsies		Tissue sections		
		Papillomas visible		ROPV DNA detected		Infected sites		
Rabbit	WPI	4 WPI	Time of culling	Infected sites	Uninfected sites	ROPV DNA in basal cells	Normal epithelium	L1/E4 protein and MCM7
A	4	Yes	Yes	5/5	5/5	–	–	–
B	4	Yes	Yes	5/5	5/5	–	–	–
C	10	Yes	No	5/5	5/5	3/4	4/4	0/4
D	10	Yes	No	5/5	5/5	–	–	–
E	15	Yes	No	5/5	1/5	2/5	5/5	0/5
F	15	Yes	No	4/5	0/5	0/5	5/5	0/5
G	19	Yes	No	5/5	1/5	2/5	5/5	0/5
H	19	Yes	No	2/5	0/5	3/4	4/4	0/4
I	22	Yes	No	4/5	0/5	6/6	6/6	0/6
J	22	Yes	No	5/5	0/5	2/8	8/8	0/8
Cont 1	14	No	No	0/5	0/5	–	–	–
Cont 2	24	No	No	0/5	0/5	–	–	–

Summary data for ROPV infected rabbits. Ten rabbits were infected with ROPV and two rabbits served as uninfected controls. Rabbits were culled at varying weeks post-infection (WPI). Rabbits were examined at 4 WPI and at the time of culling to determine whether or not papillomas were visible (columns 3 and 4). After culling, five whole tissue biopsies were analyzed from tattoo marked infected and from uninfected sites. The number of sites where ROPV DNA was detected in biopsies is shown (columns 5 and 6). A further 4–8 tattoo-marked sites were sectioned and basal cells dissected for real-time PCR analysis of ROPV DNA. The number of tattoo-marked sites from each rabbit that were positive for ROPV DNA in basal cells is shown (column 7). Adjacent sections were stained with H&E to assess pathology (column 8) and for the presence of L1, E1^E4 and MCM7 proteins (column 9). The number of sites at which viral L1 and E1^E4 proteins were detected and at which MCM7 expression was detected above the normal basal layer levels is shown.

**Table 2:** Detection of ROPV DNA in LCM samples.

Rabbit—sample no.	Control samples—copies ROPV/100 cells				
Membrane	Stroma	Epithelium	Basal cells	Upper layers
C-1	0	0	0	5.2	N/A
C-2	0	0	0	1.0	0
C-3	0	0	0	2.9	N/A
E-1	0	0	0	436.0	460455
E-2	0	0	0	192.9	N/A
G-1	0	0	0	4.9	0
G-2	0	0	0	4.7	N/A
H-1	0	0	0	25.0	0
H-2	0	0	0	96.2	0
H-4	0	0	0	631.0	0
I-1	0	0	0	2.1	0
I-3	0	0	0	9.1	0
I-4	0	0	0	1.8	0
I-6	0	0	0	8.3	0
I-8	0	0	0	17.4	0
I-9	0	0	0	4.9	0
J-1	0	0	0	11.4	0
J-3	0	0	1.6	4.7	N/A
Average	0	0	0.15	81.1	

Between four and eight basal cell samples were collected using LCM from the ROPV-infected rabbits shown in Table 1 and determination of ROPV DNA and GAPDH copy number were performed. The ROPV DNA copy number expressed per 100 cells is shown for 18 samples in which ROPV DNA was detected. For each of these sections, samples of uninfected epithelial basal cells, stromal tissue and blank slide membrane were collected. Attempts were also made to collect the upper epithelial layers above the infected basal cells although this was not always possible for technical reasons. The average ROPV DNA copy number per 100 cells is shown.

**Table 3:** 

Location	Primer/probe	Sequence	Annealing position
ROPV L1	Forward primer	CAC-TGT-CAT-GAA-CAT-TGT-CAC-AAA-G	6798–6722
ROPV L1	Reverse primer	TTG-AAA-GCA-TTG-TTA-GCC-CAA-GT	6747–6769
ROPV L1	TaqMan probe	CGA-ATG-GCA-ATG-GCG-CAG-TTG-A	6724–6745
GAPDH	Forward primer	GGA-TTT-GGC-CGC-ATT-GG	N/A
GAPDH	Reverse primer	CAA-CAT-CCA-CTT-TGC-CAG-AGT-TAA	N/A
GAPDH	TaqMan probe	CGC-CTG-GTC-ACC-AGG-GCT-GC	N/A
ROPV E1	Forward primer	CAG-TGG-GTG-GTT-TTT-AGT-GAC-TGA	1175–1198
ROPV E1	Reverse primer	CAG-TTC-CTC-AAA-CTC-ATC-GTC-AAA	1227–1250
ROPV E1	TaqMan probe	GCA-GAC-TGT-GTG-GAT-GGA-GTA-CCG-GA	1200–1225
ROPV E2	Forward primer	GGA-ATT-TGA-TCA-GGA-AAG-AGC-AA	3032–3054
ROPV E2	Reverse primer	TTC-CCA-AGC-GCC-TTA-TGC	3083–3100
ROPV E2	TaqMan probe	TTA-TAT-TGC-ACT-TTG-CCA-GAA-AAC-AT	3056–3081
ROPV E6	Forward primer	CCA-ACA-CAG-CCT-TTG-TGC-AA	567–586
ROPV E6	Reverse primer	CTA-CAG-TAA-CTA-AAG-GTG-CTG-CTT-CTG	613–639
ROPV E6	TaqMan probe	TGC-AGC-TTT-TGC-AGC-CCA-TTG-C	588–609
ROPV E7	Forward primer	TGA-GCG-TGT-GGA-TGA-TAT-AAT-TCT-G	932–956
ROPV E7	Reverse primer	CAC-ACT-CTA-CTA-CTA-TAA-GAT-AAG-CTG-AAT-GC	986–1017
ROPV E7	TaqMan probe	GAG-GAG-GAT-CAG-CAG-GGC-AAA-CAG-G	960–984
ROPV E1^E4	Forward primer	GCG-CCG-CAA-ACG-TCT-GAA-AAA-T	1123–1144
ROPV E1^E4	Reverse primer	AAC-CGG-CCC-CGA-TAA-CGA-CTT-GAC	3646–3669
